# Neurexin 1 variants as risk factors for suicide death

**DOI:** 10.1038/s41380-021-01190-2

**Published:** 2021-06-25

**Authors:** Nancy William, Carsten Reissner, Robert Sargent, Todd M. Darlington, Emily DiBlasi, Qingqin S. Li, Brooks Keeshin, William B. Callor, Elliott Ferris, Leslie Jerominski, Ken R. Smith, Erik D. Christensen, Douglas M. Gray, Nicola J. Camp, Markus Missler, Megan E. Williams, Hilary Coon

**Affiliations:** 1 Department of Psychiatry, University of Utah School of Medicine, Salt Lake City, UT USA; 2 Institute of Anatomy and Molecular Neurobiology, Westfälische Wilhelms-University, Münster, Germany; 3 Population Science, Huntsman Cancer Institute, University of Utah, Salt Lake City, UT USA; 4 Department of Psychology, University of Oregon, Eugene, OR USA; 5 Neuroscience, Janssen Research & Development, LLC, Titusville, NJ USA; 6 Department of Pediatrics, University of Utah School of Medicine, Salt Lake City, UT USA; 7 Utah State Office of the Medical Examiner, Utah Department of Health, Salt Lake City, UT USA; 8 Department of Neurobiology, University of Utah School of Medicine, Salt Lake City, UT USA; 9 Department of Internal Medicine, University of Utah School of Medicine, Salt Lake City, UT USA

**Keywords:** Neuroscience, Psychiatric disorders, Genetics

## Abstract

Suicide is a significant public health concern with complex etiology. Although the genetic component of suicide is well established, the scope of gene networks and biological mechanisms underlying suicide has yet to be defined. Previously, we reported genome-wide evidence that neurexin 1 (NRXN1), a key synapse organizing molecule, is associated with familial suicide risk. Here we present new evidence for two non-synonymous variants (*rs78540316*; P469S and *rs199784139*; H885Y) associated with increased familial risk of suicide death. We tested the impact of these variants on binding interactions with known partners and assessed functionality in a hemi-synapse formation assay. Although the formation of hemi-synapses was not altered with the P469S variant relative to wild-type, both variants increased binding to the postsynaptic binding partner, leucine-rich repeat transmembrane neuronal 2 (LRRTM2) in vitro. Our findings indicate that variants in *NRXN1* and related synaptic genes warrant further study as risk factors for suicide death.

## Introduction

Suicide is the 10th leading cause of death in the U.S and accounts for almost 800,000 deaths per year globally [1]. Suicide is preventable but its complex etiology requires further study. While the body of evidence for genes and biological mechanisms underlying suicide is growing, the genetics of suicide death are complex and remain elusive. Many researchers study suicidal behaviors, which are more common than suicide deaths [2]. However, most individuals with suicidal behaviors do not die by suicide, making the extent of shared risk factors between suicidal behaviors and suicide death uncertain [2].

There is evidence of the genetic contribution to suicide death; heritability estimates for suicide is ~50% [3, 4]. Previous studies of large, multi-generation families indicate that the risk of suicidal death extends beyond first-degree relatives [5, 6]. This extended familial risk further implicates genetic factors and not just shared environmental factors between close relatives [5].

The most widely used approach for the identification of suicide genetic risk variants is genome-wide association studies (GWAS) [7–11]. Limitations of GWAS include the genetic heterogeneity inherent in large population cohorts, which may limit statistical power for less common variants [12], and the variants identified are often merely markers for genomic locations (usually intergenic) and seldom impact gene function. Hence, there is a knowledge gap of rare, functional risk variants, which are also important to the study of complex diseases [13].

Our previous novel analyses of 215 suicide deaths in 43 extended high-risk families (7–9 generations) [6] identified genes of interest that allow us to pursue targeted follow-up of rare functional variants in an additional large, population-ascertained cohort of suicide deaths. The extended family design has increased power to detect shared genomic regions associated with risk due to the repetition of the same risk factors across many distantly related suicides. Significantly shared familial regions are highly likely to harbor variants that increase risk of suicide death. Genes in these shared regions are therefore excellent targets for follow-up in complementary designs.

**Fig. 1:**
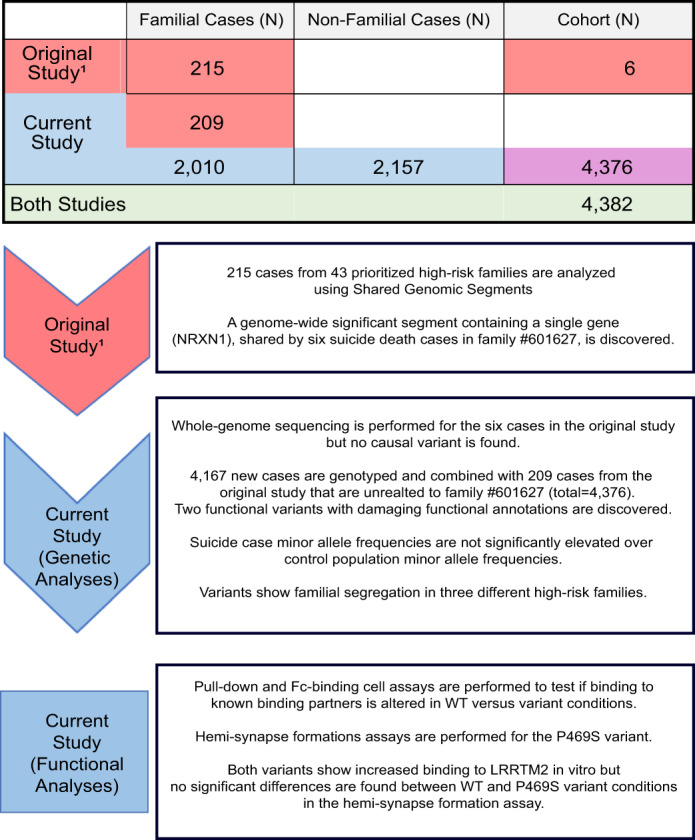
Study design. The table summarizes the number of familial and non-familial cases genotyped and analyzed in each study and serves as a reference for the study design. A total of 4382 cases were genotyped for both studies. The analysis strategy started with the prioritization of NRXN1, from a previous study of high-risk families (red arrow), led to the discovery of specific NRXN1 suicide variants (blue arrow) and finally resulted in the elucidation of their functional consequences (blue box). ^1^Coon H, Darlington T, DiBlasi E, Callor W, Ferris E, Fraser A et al. Genome-wide significant regions in 43 Utah high-risk families implicate multiple genes involved in risk for completed suicide. Mol Psychiatry. 2020;25:3077-90.

Figure 1 presents our study design. From the 30 significant familially-shared segments previously reported [6], we prioritized a genomic segment from the largest studied family (#601627), which contained a single gene-*NRXN1*. Other segments contained multiple genes, making it difficult to identify the true risk gene(s) for follow-up work. Prioritization was also based on the known functions of *NRXN1*. Synapse pathology is thought to underlie neurological and psychiatric disorders [14]. Synapse dysfunction is also an important potential target for prevention and therapeutic intervention. *NRXN1* codes for a transmembrane cell adhesion molecule that plays important roles in synaptic assembly and organization, specification, neurotransmission, and synaptic plasticity in both inhibitory and excitatory synapses [15–18]. *NRXN1* can also be prioritized due to its psychiatric relevance, highlighted in a recent review by Hu et al. [19]. Previous studies showed that several missense and structural variations in *NRXN1* are associated with autism, schizophrenia, developmental abnormalities, and psychiatric drug response [16, 19–27]. There is also evidence that brain expression of *NRXN1* is altered in individuals with bipolar disorder or schizophrenia who died by suicide [28, 29]. A synaptic binding partner of NRXN1, LRRTM4, is also implicated in increased suicide risk [30, 31]. Few studies have tested the functional consequences of specific disease-associated *NRXN1* variants. Variants found in autism and schizophrenia were tested in human-induced neurons and resulted in alterations in calcium signaling, impairment of synaptic function, and changes in cell fate [32–34].

In this study, our objectives were to identify *NRXN1* variants, in our suicide death cohort, with likely functional consequences on the gene and test their function. We used whole-genome sequencing to search for likely causal functional variants in *NRXN1* shared across the six related suicides in the original study [6], supporting the genome-wide significant result. Additionally, we screened genotyping data from 4376 other Utah suicide deaths in our cohort, excluding the six original familial cases,  to identify other functional *NRXN1* variants. We aimed to identify *NRXN1* variants that were at a sufficient frequency in suicide cases to warrant functional analysis (frequency of at least 0.001) and that were rare (frequency < 0.01) in population controls. We also used our suicide death sample to identify functional *NRXN1* variants that segregated in other identified high-risk suicide families. To accomplish this analysis, we used genealogical data from a total of 241 ascertained high-risk families. These 241 high-risk families include the 43 described in our previous study and include approximately half of the 4376 genotyped cases (2219 cases) in our suicide death cohort. This approach allowed us to target two functional *NRXN1* variants with additional evidence of familial transmission. Finally, we tested the functional consequences of the two identified *NRXN1* suicide variants on NRXN1 binding interactions to known partners and in hemi-synapse formation.

## Materials/subjects and methods

### The Utah suicide research resource

In collaboration with the Utah State Office of the Medical Examiner (OME), and with Institutional Review Board (IRB) permissions from the University of Utah, Utah Department of Health, and Intermountain Healthcare, we have been collecting de-identified DNA samples from suicide deaths since 1997. Suicide status is conservatively determined by the OME based on investigation of the scene of death, interviews with kin and friends of the deceased, review of medical and public records concerning the individual, and autopsy and toxicology reports. Suicide deaths are securely linked to medical information and family records via the Utah Population Database (UPDB), a data resource with state-wide demographic and health information, and with extended genealogical records [35].

Genealogical data allowed the determination of high-risk families through the comparison of the total number of suicide deaths in the family vs. the expected number in the family, accounting for known population rates of suicide death [6]. Our previous study prioritized 43 of the highest-risk families containing 215 suicide deaths [6]. However, in our data resource, a total of 241 high-risk families that meet a familial significance threshold of *p* < 0.01 have now been ascertained. These families include 2219 Utah suicide deaths with genotyping data (50.71% of the sample). The genealogical information in our data resource not only allowed for prioritization of specific regions, including the region with *NRXN1* that is the focus of the follow-up, but the information additionally allowed for prioritization of single functional variants that show familial transmission.

Psychiatric diagnoses on Utah suicide deaths from electronic medical record (EMR) data are available for ~80% of suicide deaths with genotyping data. The Utah suicide research sample is population-based and reflects suicide deaths that crosscut diagnoses.

Our data also allows for testing background polygenic risk, through the derivation of polygenic risk scores (PRS) [7]. We tested for *NRXN1* variant carrier vs. non-carrier differences in polygenic risks for a subset of diagnostic and behavioral traits hypothesized to have *NRXN1* associations: suicide attempt, major depressive disorder, depressive symptoms, schizophrenia, autism, bipolar disorder, anxiety disorder, attention deficit hyperactivity disorder, alcohol abuse, smoking, post-traumatic stress disorder, neuroticism, and extraversion. Polygenic risk scores for these traits had already been computed for our GWAS study [7]. To test for carrier vs. non-carrier differences, we used logistic regression, adjusting for genetically determined ancestry principal components derived as part of our GWAS [7].

### Genotyping

In total, 4382 cases (215 in the previously analyzed high-risk extended families and 4167 additional cases) were genotyped using the genome-wide Illumina PsychArray, an array with 265,000 tag single nucleotide polymorphisms (SNPs), 245,000 markers from Illumina Exome BeadChip, and 50,000 additional markers with clinical or psychiatric relevance [36]. The SNPs were aligned in the forward strand HG19 configuration by comparing the SNP calls within the 1000 Genomes Project [37]. SNPs were removed if the forward strand status was unclear, or if variants were not polymorphic, and non-autosomal. Additional quality control of genotyping was done using PLINK [38], as previously described [6].

### Sequencing

To search for potentially causal risk variants in the 0.92 Mb prioritized candidate region containing *NRXN1*, we generated sequence data for the six suicide deaths in the previously studied family that shared this region (#601627). Whole-genome sequencing was performed on cases using Illumina NGS technology [39] using DNA from blood at 30x-60x coverage. We used BWA-MEM [40] for mapping sequencing reads to the human genome reference sequence and for variant detection and calling, we used best practice GATK-based procedures [41].

### Genetic analyses

We used the GEMINI software tool [42] to search for causal single nucleotide functional variants directly shared in family #601627, and the LUMPY tool [43] to search for shared structural variants. Single nucleotide variants of interest were defined as those in the 0.92 Mb shared region where the minor allele was present in all six cases, the minor allele frequency was <0.01 in 525 Utah controls and publicly-available data (gnomAD; 44), and the variant had medium or high impact severity annotations. The 525 Utah controls used in this analysis included 160 unrelated Utah individuals selected for the absence of disease who were in the CEPH genetic mapping sample [44], 78 unrelated healthy Utahns over age 90 from a study of extreme longevity [45], and 287 1000 Genomes Utah samples [37]. We used SIFT (sorting intolerant from tolerant; [46]) and PolyPhen (polymorphism phenotyping; [47]) to determine the predicted functional consequences of the missense variants we discovered. The algorithms for these computational tools are based on variables such as whether the variants occur in conserved regions, the physical and chemical properties of the amino acids involved, and the structural properties of a protein. All amino acid substitutions across the proteome are assessed to determine which mutations are likely to result in pathogenicity. We searched for additional evidence of *NRXN1* functional risk variants using 4376 additional Utah suicides with Illumina PsychArray genotyping. These analyses included 209 cases in the original extended family study who were not related to family #601627 and 4167 new Utah cases. Inclusion of the 209 original cases is justified because this analysis is focused on single *NRXN1* functional variants which could occur on a sufficiently small haplotype to escape detection in our original familial analysis, or variants may be shared across too few cases within an extended family to be detected in the original analysis. We analyzed functional *NRXN1* variants that spanned the entire gene, comparing to frequencies in Non-Finnish European control samples from 1000 Genomes and gnomAD. Because our original evidence suggested that *NRXN1* may be associated with familial suicide risk, we not only tested for case-control frequency differences but also used our extensive genealogical data to estimate the nominal probability of familial segregation of single functional variants. Segregation probabilities were computed by considering variant frequency, number of meioses between sharing cases, and the chance of introduction of the variant through a marry-in spouse vs. transmission at each generation (see Supplementary Methods, and Supplementary Fig. 1 for details). Variants showing significant familial segregation with suicide death were pursued for further functional analysis.

### Plasmids

**Fig. 2:**
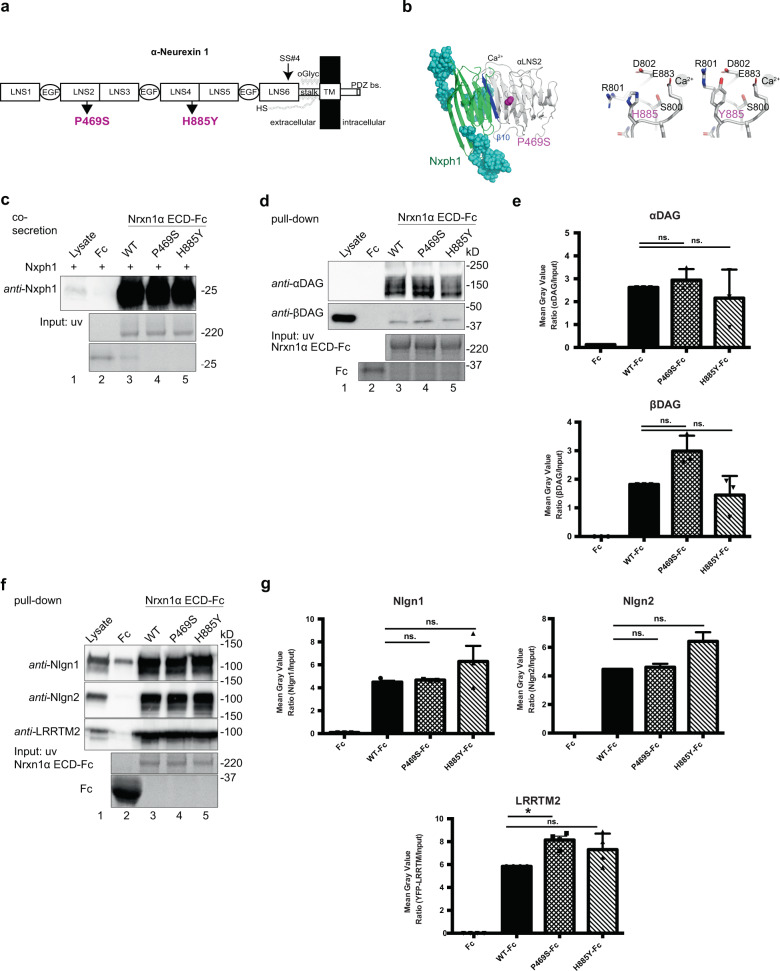
P469S variant shows increased binding to LRRTM2 in pull-down assay. **a** Schematic of Nrxn1α protein lacking insert at splice site #4 (SS#4), including locations of the suicide-associated variants identified in this study (magenta). Laminin neurexin sex-hormone binding globulin domain (LNS); epidermal growth factor-like domain (EGF); transmembrane region (TM); O-glycosylation sequence (oGlyc); heparan sulfate (HS); PDZ bs.; PSD-95/Dlg/ZO-1 binding site. **b** In the left panel, ribbon diagram of variant P469S (magenta) in the αLNS2 domain, and binding of Nxph1 (green). In the right panel, stick model of variant H885Y, proximal to the Ca^2+^-binding site at the LNS4 domain. **c** Western blot of Nrxn1α and Nxph1 pull-downs. Note that neither variant alters binding. **d** Western blot of pull-down of α-DAG and β-DAG with the extracellular Fc-tagged domain of Nrxn1α shows undisturbed DAG binding with wild-type and variant Nrxn1α. **e** Quantification of 2d (*n* = 3); n.s. indicates not significant by one-way ANOVA. **f** Western blot pull-down shows Nrxn1α variants bind Nlgn1 and Nlgn2 at similar levels to WT Nrxn1α but the P469S variant binds YFP-LRRTM2 at increased levels to WT Nrxn1α. **g** Quantification of 2f (*n* = 3); One-way ANOVA using GraphPad Prism followed by pairwise posttests. *p < 0.05, n.s. indicates not significant.

N-terminally myc-tagged LRRTM2-4 expression constructs were previously described [48] and gifted by Dr. Joris de Wit (Katholieke Universiteit Leuven, Belgium). pCAG-HA-rat Nrxn1α, lacking alternatively spliced segment 4 (-AS4), was generated by Dr. Peter Scheiffele [49] and obtained from Addgene (Addgene plasmid # 58266; http://n2t.net/addgene:58266; RRID:Addgene_58266). To generate the wild-type HA-Nrxn1α-ecto-Fc construct, the HA-rat-Nrxn1α extracellular domain was PCR amplified from the pCAG-HA-ratNrxn1α (-AS4) construct and subcloned to be in frame with pTEV-Fc containing a human IgG Fc. To generate single variant Fc (P469S or H885Y) and double mutated (P469S/D1216A or H885Y/D1216A) Fc constructs, we used site-directed-mutagenesis (Q5^®^ Site-Directed Mutagenesis Kit, New England Biolabs) and constructs were verified by sequencing. The D1216A mutation prevents Ca^2+^-binding at laminin-neurexin-sex hormone-binding globulin domain 6 (LNS6). For full-length Nrxn1α variant constructs, similar mutagenesis procedures were performed, and the full-length Nrxn1α (-AS4) regions were then subcloned back into the original pCAG vector using standard PCR cloning and verified by sequencing. Plasmids for rat neurexophilin 1 (Nxph1; pCMVD2), dystroglycan (DAG; pCMVDAG), neuroligin 1 (Nlgn1; pCMVNL1-B), neuroligin 2 (Nlgn2; pCMVNL2-1) and for like-acetyl-glucosaminyl-transferase (LARGE; pCMV6-XL4 LARGE), have been previously described [50]. Full-length YFP-LRRTM2 used in Fig. 2f was a gift from Fredrik Sterky (SciLifeLab, Stockholm, Sweden).

### Cell culture

Cell cultures were kept in a humidified incubator and maintained at 37° C and 5% CO_2_. Cell lines used: Chinese Hamster Ovary (CHO-K1) cells (RRID: CVCL_0214, female) were grown in F12K media (Life Technologies, Carlsbad, CA, United States), 10% FBS (Fetal Bovine Serum) (Life Technologies), and penicillin/streptomycin; HEK293 cells (RRID: CVCL_0063, female) were grown in DMEM (Life Technologies), 10% FBS, and penicillin/streptomycin. Cells were transfected using polyethylenimine (PEI, Polysciences, Warrington, PA, United States) at a ratio of 10 mg PEI/1mg DNA for CHO cells, 4 mg PEI/1mg DNA for 293 cells used in the expression of Fc proteins, and 10 mg PEI/1mg DNA for 293 cells transfected for the synapse formation assay.

Neurons: preparation of hippocampal rat cultures were previously described [51, 52]. Briefly, P0 rat cortical glia were cultured to form monolayers on Poly-D-Lysine/collagen-coated coverslips in 24-well plates. P0 rat hippocampi from Sprague Dawley rats of both sexes (RRID: RGD_734476) were dissected one week later in cold 4-(2-hydroxyethyl)-1-piperazinee-thanesulfonic acid (HEPES)-buffered saline solution (Life Technologies), treated with enzymatic papain for 30 min, dissociated, and plated onto glial monolayers at a density of 5 × 10^4^ cells/well.

### Fc-binding assay

Wild-type (WT), P469S, and H885Y HA-Nrxn1α-ecto-Fc constructs were transfected into HEK293 cells using PEI and proteins were extracted from the media based on previously described methods [51]. Briefly, transfected cells were incubated in OptiMEM (Life Technologies) media for 5 days. Then, the conditioned media was harvested, concentrated, and filtered using 100 kD Amicon Ultra filter units (Millipore, Billerica, MA, United States). Fc protein concentrations were estimated by Western blot (Supplementary Fig. 2a) using known concentrations of purified human Fc (Jackson ImmunoResearch, West Grove, PA, United States) as a standard. Fc proteins were run on 8% SDS-PAGE gels and transferred to nitrocellulose membranes using the iBlot system (Life Technologies). Membranes were incubated in blocking solution (50 mM Tris pH 7.5, 300 mM NaCl, 3% wt/vol dry milk powder, and 0.05% Tween-20) for 1 h, and, incubated in HRP-conjugated secondary antibody (Jackson ImmunoResearch) for 1 h at room temperature. The Bio-Rad Clarity ECL kit on a Bio-Rad ChemiDoc XRS+ imaging system was used for protein detection. Nrxn1α WT-ecto-Fc, Nrxn1α variant-ecto-Fc, and control-Fc (at ~1 μg/ml concentrations) were then added to GFP-only and LRRTM + GFP co-expressing CHO cells 24 h after transfection with PEI. Cells were incubated on ice for 30 min to facilitate binding of the Fc proteins. Following Fc treatment, the cells were washed gently with 1x Phosphate Buffered Saline (PBS) (Life Technologies) and fixed with 4% paraformaldehyde (PFA).

### Biochemical procedures and pull-down assays

DNA constructs coding for soluble ecto-Fc of WT or mutated Nrxn1α, were transfected into HEK293 cells using calcium phosphate as described previously [50]. Similarly, co-secreted complexes of Nxph1 with WT or variant Nrxn1-Fc were harvested after calcium phosphate co-transfection of constructs. To obtain recombinant full-length αβDAG, DNA constructs coding for αβDAG and LARGE were co-transfected in N2A cells using calcium phosphate, and cells were lysed 72 h after transfection using 1% Triton in Tris-buffer (50 mM Tris pH 7.5, 80 mM NaCl, 5 mM CaCl2, protease inhibitor cocktail III, Merck) for 30 min at 4 °C. After centrifugation (1 min, 21,000 × *g*, 4 °C) supernatant was used immediately or frozen by liquid nitrogen and stored at −80 °C. Full-length Nlgn1, Nlgn2, or LRRTM2 were expressed in COS7 cells using DEAE-dextran transfection, based on previously described methods [50] and cells were incubated for 2 days before lysis using 1% Triton in Tris-buffer, as described above.

For pull-down assays, Protein A-conjugated Sepharose beads were added to either media containing co-secreted Nxph1/Nrxn1α-Fc complexes or cell lysates, incubated overnight, washed three times, and analyzed directly. N2A lysates with DAG were incubated with Nrxn1α-bound Protein A-beads overnight in the presence of 5mM CaCl_2_. Similarly, COS lysates containing either Nlgn1, Nlgn2, or YFP-LRRTM2 were also incubated with Nrxn1α-bound Protein A-beads overnight in the presence of 5mM CaCl_2_. Complexes were centrifugated (30 s, 11,000 × *g*, 4 °C), washed three times, boiled for 5 min in sample buffer, and analyzed by Western Blot using stain-free 4–15% gradient gels and the Mini-Protean system (Bio-Rad, Feldkirchen, Germany). Gray values of each blot have been normalized to input values which were detected by UV using a stain-free gel (Bio-Rad), anti-Fc antibody (I9135, Sigma), and anti-HA antibody (16B12, Biolegend) for HA-Nrxn1a-Fc variants (*n* = 3). Intensities have been measured using ImageJ and analyzed in a one-way ANOVA using GraphPad Prism.

### Immunocytochemistry

Cells were fixed in 4% PFA for 10 min and washed three times with 1x PBS. Cells were blocked for 30 min in blocking buffer (1x PBS + 3%BSA + 0.1% Triton-X100). Primary antibodies were diluted in blocking buffer without Triton-X100 and cells were incubated for 2 h at room temperature with the primary antibodies. The following primary antibodies were used: goat anti-GFP 1: 3000, (RRID:AB_305643, Abcam, Cambridge, MA, United States), rabbit anti-myc 1:1000 (RRID:AB_439680, Sigma Aldrich, St. Louis, MO, United States), chicken anti-MAP2 1:5000 (RRID:AB_2138153, Abcam), guinea pig anti-vGLUT1 1:2000 (RRID:AB_2301751, Millipore), mouse anti-PSD-95 1:1000 (RRID:AB_2315909, NeuroMab, UC Davis/NIH NeuroMAB Facility, Davis, CA, United States), guinea pig anti-vGAT 1:1000 (RRID: AB_212625, Millipore), mouse anti-gephyrin 1:1000 (RRID:AB_2619837, Synaptic Systems, Goettingen, Germany). After incubation with primary antibodies, cells were washed three times and incubated in the dark with secondary antibodies for 1 h, at room temperature. Cy3-conjugated donkey anti-human IgG 1:1000 (RRID:AB_2340534, Jackson ImmunoResearch) was used to visualize Fc proteins. All secondary antibodies were donkey-derived and diluted 1:1000 (Jackson ImmunoResearch) and included, donkey anti-chicken-DyLight 405 (RRID:AB_2340373), donkey anti-goat-Alexa 488 (RRID:AB_2336933), donkey anti-guinea pig Alexa 647 (RRID: AB_2340476), and donkey anti-rabbit Alexa 647 (RRID: AB_2492288). Slides were prepared for imaging with Fluoromount-G (Southern Biotech, Birmingham, AL, United States).

### Hemi-synapse formation assay

Mixed-culture assays for hemi-synapse formation were performed based on standard methods previously described [53]. Briefly, HEK293 cells were transfected, using PEI, with GFP-only, GFP+ WT full-length HA-Nrxn1α, GFP + P469S HA-Nrxn1α, and GFP+ H885Y full-length HA-Nrxn1α constructs. 24 hours later, the transfected HEK293 cells were washed and dissociated in neuronal feeding media and seeded at 3 × 10^4^ cells/well in a 24-well plate of primary neuron cultures grown on a glia monolayer (DIV 10). The co-cultures were incubated for 36 h and fixed on DIV 12 in 1x PBS + 4% PFA for 10 min at room temperature.

### Confocal imaging and analysis

Z-stack images were captured using a Zeiss LSM 710 confocal microscope at 40x to observe Fc binding in CHO cells. The maximum intensity projection of the Z-stack was analyzed using NIH ImageJ software under blind conditions. For the Fc-binding assay analysis, CHO cells were traced in the 647 nm channel based on myc-LRRTM immunoreactivity. The traced cell area selection was expanded by 1 µm to establish an ROI for each cell. The ROI area and mean-gray values (MGV) were then measured. The MGV and ROI areas of each respective cell were also measured in the 555 nm channel, which detected Fc immunoreactivity. To account for expression differences in the CHO cells, the ratio of the MGV of both channels was calculated (Fc MGV/LRRTM MGV). Values across all conditions were analyzed using a one-way ANOVA analysis in GraphPad Prism software.

For the mixed co-culture assay, HEK293 cells were imaged at 63x and chosen for further analysis if MAP2 staining (405 nm channel) revealed at least one dendrite crossing or touching a GFP-positive HEK293 cell (488 nm channel). Images were de-speckled and optimal thresholds were determined for PSD-95 (555 nm) and vGLUT1 (647 nm) channels, for excitatory synapses, and gephyrin (555 nm) and vGAT channels (647 nm) for inhibitory synapses. The GFP cell filler channel (488 nm) was thresholded and the selected area of a cell was enlarged by 0.5 µm; this selection became the cell ROI, and cell ROI area measurements were recorded. In addition, the remaining channels were thresholded and particle count and particle area measurement were taken for puncta in the 555 nm and 647 nm channels. A constant particle parameter of 0.2–0.8 µm was imposed to define puncta. vGLUT or vGAT puncta that overlapped with PSD-95 and gephyrin puncta respectively were subtracted from the images to remove false-positive neuron-neuron synapses. The puncta count and puncta area of PSD-95-only and gephyrin-only puncta were recorded and indicated induced hemi-synapses. A one-way ANOVA analysis based on the area of hemi-synapse puncta was used for comparison across all conditions in GraphPad Prism software.

## Results

### NRXN1 sequence variants in six familial cases

From our previous high-risk family study [6], family #601627 showed significant sharing for a chromosome 2 genomic segment containing the single gene *NRXN1*. The familial analysis method used in this previous study implicated this target gene but was not designed to identify specific risk variants. Therefore, we searched for causal single-nucleotide and structural variants in *NRXN1* directly shared across the six suicide deaths in this original high-risk family using whole-genome sequence data. We did not discover any obvious rare, medium, or high impact causal or structural variants shared by these six familial cases indicating regulatory variant(s) must be responsible for the significance in this extended family.

### Functional NRXN1 variants from genotyping

Though direct evidence in the discovery high-risk family was not apparent, the familial genome-wide significant result implicating *NRXN1* was sufficiently strong to warrant a search for additional risk variants in our full suicide research cohort. Therefore, we analyzed 4376 additional cases that were genotyped on the Illumina PsychArray platform, retaining protein-coding variants in NRXN1. We found two non-synonymous variants where both Polyphen [47] and SIFT [46] annotations indicated likely functional effects, and the minor allele was observed in at least five suicides (frequency of at least 0.001). These variants are GRCh37 2-50847195-G-A; *rs78540316*; P469S and GRCh37 2-50724817-G-A; *rs199784139*; H885Y, with annotations for “possibly” and “probably” damaging in Polyphen and “deleterious” in SIFT. Twenty-eight suicide cases were heterozygous for P469S (frequency: 28 / 8752 = 0.0032); the 1000Genomes European frequency of this variant is 29 / 8758 = 0.0033 and 413 / 126558 = 0.0033 in the gnomAD [54] European non-Finnish population. For H885Y, 10 suicide cases were heterozygous (frequency: 10 / 8752 = 0.0011); the 1000 Genomes European frequency of this variant is 11 / 8762 = 0.0013, and the frequency in gnomAD European non-Finnish controls is 134 / 127740 = 0.0011. One case in our cohort carried both risk variants. While the suicide case frequencies were not significantly elevated over population control frequencies in a case-control design, both *NRXN1* variants showed familial segregation in extended families with familial risk of *p* < 0.01. Segregation of P469S occurred in two different high-risk families: one with seven meioses separating the cases, and the other with 11 meioses separating the cases. Segregation of H885Y occurred in one other high-risk family, separated by 12 meioses (see Supplementary Methods, and Supplementary Fig. 1).

### Post hoc diagnostic and polygenic risk characterization of suicide deaths with the NRXN1 variants

Cases with the two *NRXN1* variants that are the focus of this study had diagnoses across 27 different phenotypic clusters related to psychiatric and medical co-morbidities (Supplementary Table 1). Of the 38 cases with these variants, 19 had evidence of at least one other medical or psychiatric diagnosis. However, no unusual clustering of phenotypes was observed relative to what was observed in our suicide cohort without variants, who had records for diagnoses (2722 cases). The most frequent diagnoses among all cases were chronic pain (15 cases), depression (12 cases), and anxiety (9 cases). Bipolar diagnoses were found in five cases.

Tests for *NRXN1* variant carrier vs. non-carrier differences among the polygenic risk scores for suicide attempt, major depressive disorder, depressive symptoms, schizophrenia, autism, bipolar disorder, anxiety disorder, attention deficit hyperactivity disorder, alcohol abuse, smoking, post-traumatic stress disorder, neuroticism, and extraversion did not result in any nominal significant differences.

### Suicide-associated variants show increased binding to LRRTM2 in vitro

We next tested whether either *rs78540316* (P469S) or *rs199784139* (H885Y) altered Nrxn1 function. There are multiple isoforms of Nrxn1, and the variants we identified alter residues in the LNS2 and LNS4 extracellular domains of the α-isoform (Fig. 2a). The extracellular domain is necessary for the binding of Nrxn1 to its many ligands [18, 50]. Thus, we tested the ability of each variant to bind to several Nrxn1α ligands using pull-down assays. Ligands tested included neurexophilin 1 (Nxph1), the dystroglycan complex (DAG), leucine rich repeat transmembrane neuronal 2 (LRRTM2), neuroligin 1 (Nlgn1), and neuroligin 2 (Nlgn2).

Nxph1, a glycoprotein secreted by subpopulations of neurons [50], forms a complex with the extracellular LNS2 domain of Nrxn1α (Fig. 2b, left) in the secretory pathway of cells, and co-secretes into media [50]. Therefore, to test Nxph1 binding with Nrxn1α variants, we co-expressed Nxph1 with the extracellular domain of Nrxn1α fused to an Fc tag in HEK293 cells. The media was collected and the co-secreted recombinant Nxph1/Nrxn1α-ecto-Fc complex was bound to Protein A-beads for western blot analysis (Fig. 2c). Our results indicate that both variants bound to Nxph1 similar to WT (Fig. 2c). This suggests that neither *NRXN1* variant interferes with secretion and binding to Nxph1.

The extracellular domain of Nrxn1 also binds the alpha subunit of the adhesion receptor protein DAG ([50]; Supplementary Fig. 3a). α-DAG directly interacts with residues in the LNS2 and LNS6 domains of Nrxn1α and physically covers the intervening LNS3, LNS4, and LNS5 domains (Supplementary Fig. 3a). We reasoned that the suicide-associated Nrxn1α variants could interfere with α-DAG binding because the P469S mutation lies in the LNS2 domain (Fig. 2b, left) and the H885Y mutation is proximal to the Ca^2+^ binding site within the LNS4 domain of Nrxn1α (Fig. 2b, right). To test DAG binding with suicide-associated Nrxn1α variants, we expressed the DAG complex in N2A cells along with its glycosylating co-factor LARGE, which is necessary for LNS domain binding [50]. We then used Nrxn1α-ecto-Fc pre-bound to Protein A beads as bait to pull down DAG from N2A cell lysates. Western blot analysis of the precipitate revealed that each Nrxn1α variant and WT Nrxn1α bound to DAG at similar levels (Fig. 2d–e). Because DAG binds Nrxn1 in multiple locations, we also tested if weakening the interaction between Nrxn1α and DAG would reveal compromised binding with either of the suicide-associated variants. We did this by combining each variant with a D1216A mutation in Nrxn1α. This mutation prevents DAG from interacting with the LNS6 domain. However, combining the D1216A mutation with either suicide-associated variant did not prevent binding of α-DAG compared to controls (Supplementary Fig. 3b).

Next, we tested binding of Nrxn1α to its trans-synaptic receptors, Nlgn1, Nlgn2 and LRRTM2 (Supplementary Fig. 3c). We did this by binding purified Nrxn1α-ecto-Fc proteins to Protein A beads and incubating them with lysates prepared from COS7 cells expressing each receptor. We found no influence of either suicide-associated variant on binding to Nlgn1 or Nlgn2, but we observed that the P469S variant resulted in increased binding to the LRRTM2 receptor (Fig. 2f–g). In summary, binding of purified Nrxn1α variants to all the ligands tested was similar to WT Nrxn1α binding, with the exception of variant P469S binding to LRRTM2, which was increased relative to WT Nrxn1α binding.

**Fig. 3:**
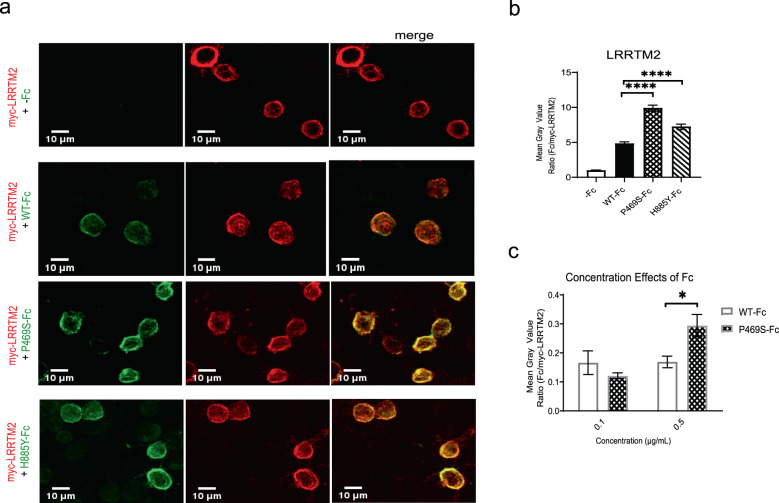
Suicide variants show increased binding to LRRTM2 in vitro. **a** Representative confocal images of Fc cell surface binding assays. **b**-**c** Quantification of Fc-binding assays, normalized to control-Fc. **b** Quantification of experiments using 1.0 ug/mL concentration of Fc proteins. **c** Quantification of the same assay but using 0.1 and 0.5 ug/mL of P469S-Fc only. (****p < 0.0001, One-way ANOVA with Dunnett’s T3 multiple comparisons tests, *p = 0.02, multiple *t* tests; *n* = 39 (WT), *n* = 48 (P469S), *n* = 37 (H885Y) cells from three different cultures; ±SEM reported).

We further tested if the suicide-associated neurexin variants altered binding between Nrxn1 and the LRRTM family using a cell-based assay. This in vitro assay used shorter incubation times than those used in the biochemical assays and a different environmental milieu since these factors could influence binding. Previous studies have shown that LRRTM1 is poorly expressed on the surface of non-neuronal cells [48] so we tested Nrxn1α binding to LRRTM2, 3, and 4. We treated CHO cells expressing myc-tagged LRRTMs with conditioned media containing WT and variant Nrxn1α-Fc proteins (Supplementary Fig. 2a) and Fc-binding was detected by immunofluorescence (Fig. 3a, Supplementary Fig 2b).

Interestingly, we found that both suicide-associated variants had significantly increased binding to LRRTM2 in vitro compared to WT with P469S showing a two-fold increase in binding to LRRTM2 (Fig. 3b). The other conditions tested showed no change from WT (Supplementary Fig. 2c). To determine whether the concentration of Nrxn1α ecto-Fc had any effect on its ability to interact with LRRTM2, we repeated this experiment using one-half and one-tenth the original concentration of Nrxn1α ecto-Fc (Fig. 3c). We observed enhanced binding of Nrxn1α ecto-Fc to LRRTM2 at 0.5 µg/mL (half the concentration used in the initial binding assay), but the effect was diminished at 0.1 ug/mL.

### P469S variant induces hemi-synapses similar to WT in vitro

**Fig. 4:**
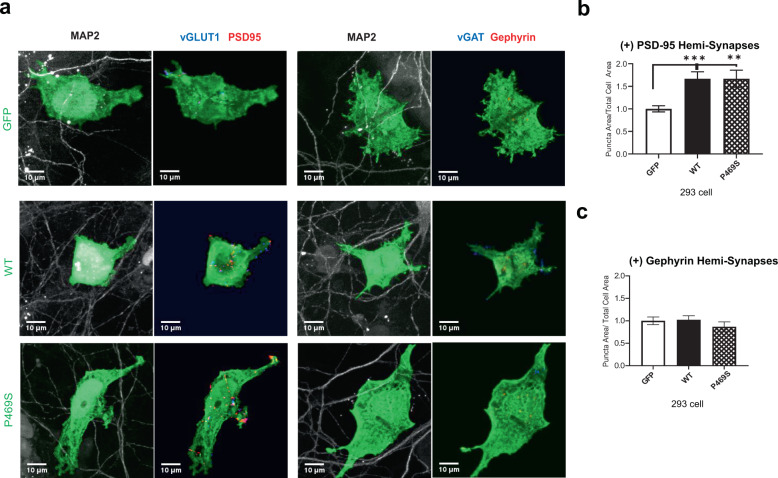
P469S variant induces hemi-synapses similar to WT in vitro. **a** Representative confocal images of the hemi-synapse assay. 293 cells were transfected with GFP (control) or co-transfected with full-length WT+GFP and variant+GFP constructs. MAP2 staining (white) indicates dendrites. For excitatory synapses, presynaptic vGLUT1 (blue) and postsynaptic PSD-95 (red) markers were used. For inhibitory synapses, presynaptic vGAT (blue) and postsynaptic gephyrin (red) markers were used. **b**-**c** Quantification of the excitatory and inhibitory hemi-synapse assays. **b** P469S variant induces excitatory synapses similar to WT(***p = 0.0004, **p = 0.0036, One-way ANOVA analysis with Dunnett’s T3 multiple comparisons tests, ns *p* > 0.05 for all other comparisons; *n* = 41 [GFP], *n* = 33 [WT], *n* = 29 [P469S] cells from three different cultures; ±SEM reported). **c** Nrxn1α does not induce inhibitory synapses and variants do not change its activity (*n* = 46 [GFP], *n* = 50 [WT], *n* = 27 [P469S] cells from three different cultures; ±SEM reported); no significant differences by one-way ANOVA.

Given that variant P469S showed the strongest increase in binding with LRRTM2, we tested whether this variant had altered hemi-synaptogenic properties compared to WT. Previous studies showed that when non-neuronal cells expressing Nrxn1α (-AS4) are plated with neurons, the neurons will form postsynaptic specializations or hemi-synapses in response to Nrxn1α-expressing cells [53, 55, 56]. Thus, we compared hemi-synapse formation induced by WT Nrxn1α and the P469S variant. Newly formed hemi-synapses between HEK293 cells and neurons were defined by the presence of the excitatory postsynaptic marker PSD-95 or the inhibitory postsynaptic marker gephyrin (Fig. 4a). Although we observed an induction of excitatory synapses (Fig. 4b), we did not observe differences between WT Nrxn1α and the P469S variant in the formation of hemi-synapses (Fig. 4b–c).

## Discussion

High-risk families are insightful data resources for genetic risk discovery. From previous results of extended high-risk families [6], we prioritized *NRXN1* as a target for studies of genetic risk for familial suicide. Subsequent follow-up in 4376 additional suicide deaths identified two functional *NRXN1* missense variants with evidence of familial association with suicide. These variants showed increased binding of the Nrxn1 receptor LRRTM2 in vitro. Though more work must be done under physiological conditions, our results provide functional evidence that these variants could alter synaptic signaling.

### NRXN1 is a suicide risk factor

*NRXN1* and its interacting genes are compelling candidates for suicide risk, as they are associated with a broad spectrum of neurological and psychiatric disorders (autism, schizophrenia, anxiety, depression, bipolar disorder, and attention deficit hyperactive disorder) [17, 19, 25, 57–59]. This evidence suggests the importance of *NRXN1* to affect multiple manifestations of psychopathology, mirroring suicide risk, which also crosscuts psychiatric diagnoses [5]. The suicide cases carrying the variants we tested in this study exhibited a range of diagnoses, suggesting the two *NRXN1* variants may be related to suicide risk that is not confined to a specific psychiatric or clinical subpopulation.

### Functional implications of suicide risk variants

*NRXN1* has known functional roles specifically at the synapse in regulating both excitation, and inhibition in the brain [50, 55, 59–62]. We showed that two *NRXN1* variants associated with familial suicide risk resulted in increased binding of LRRTM2, suggesting that synapse regulation may be important in risk. Although our hemi-synapse formation assays did not result in changes in the overall number of synapses, more subtle changes in types of synapses or synapse activity may be key and could dramatically impact neural behavior. Indeed, differences in neuronal distributions of neurexin isoforms and their interacting proteins in the brain are important in neural circuity [15, 50, 62, 63]. Interestingly, we observed an increase, rather than a decrease in binding to LRRTM2. A similar increased binding of LRRTM2 to Nrxn3α has recently been found for an A687T SNP variant related to intellectual disability and epilepsy, resulting in a gain-of-function presynaptic phenotype at excitatory synapses [64]. Ultimately, generation of in vivo models, such as knock-in mouse mutants [64] will be necessary to uncover the functional consequences of the variants in this study.

Previous studies showed that levels of Nrxn1α are important for LRRTM2-mediated excitatory presynaptic differentiation [48]. The NRXN-LRRTM complex was a target in drug-screening for inhibitors because of its link to neurological disorders [65]. Our synapse formation assays did not specifically assess LRRTM2-Nrxn1 synapses, so future work could be directed toward investigating these specific synapses. Finally, *NRXN1* may have other unknown functions that are impacted by these variants which were not tested. The dynamics of *NRXN1* and binding partners are complex and may additionally be affected by biological processes that exist in vivo [50, 55, 60, 66, 67].

### Nature and impact of suicide risk variants

We observed a lack of elevation in allele frequencies of our variants in our full suicide cohort compared to controls, but also observed their presence in other high-risk families. Together, these observations suggest a small effect size of the variants. We acknowledge that effects may be magnified or diminished by the presence/absence of other moderating factors, including other rare variants and/or underlying polygenic risks of psychiatric diagnoses or traits. Post hoc analyses of diagnostic phenotypes did not show unusual clustering of any co-occurring condition among the suicides that were carriers of the two *NRXN1* variants compared to non-carrier suicides. In addition, we did not find significant carrier vs. non-carrier differences in polygenic risk scores for selected psychiatric diagnoses. However, we note that we are powered only to observe large differences in phenotypic and polygenic risk effects.

Although we prioritized functional protein-coding variants, allowing the study of mechanism of action, it is also possible that variants in regulatory regions and haplotypes are important for risk, and should be considered in future studies. In a complex phenotype such as suicide, family designs may help identify variants that elevate, rather than drive the risk for suicide. There may be subtle alterations in the regulation of the gene in question, accompanied by additional evidence for the occurrence of rare functional coding variants in that gene in a small minority of cases. Interestingly, a similar scenario was recently described in a familial discovery in the cancer literature [68]. In addition, there is evidence *NRXN1* may be proximally and distally regulated [69] and *NRXN1* variants can regulate other functionally relevant genes. For example, using the rSNPBase database [70], we discovered intronic *NRXN1* variants associated with expression quantitative trait loci genes such as Rho GTPase activating protein 26 (*ARHGAP26*), which is differentially expressed in the dorsolateral prefrontal cortex between suicide and non-suicide mood disorder subjects [71]. In addition, we note that other familially shared suggestive regulatory genomic segments in our original family study [6] contained genes encoding for proteins that interact with NRXN1 at synapses (*LRRTM3*, *NXPH1*) [50, 67], suggesting a potentially more complex role of *NRXN1*-related synapse pathology in suicide risk. Future studies should also consider a broader pathway-based approach. In conclusion, our findings suggest changes in the *NRXN1* gene involving synaptic dynamics may contribute to increased suicide risk. Suicide risk is complex. Genetic changes may elevate risk but are not alone sufficient to cause suicide in the absence of complex interactions with many other genetic and environmental risk factors.

Supplementary information is available at MP’s website.

### Data sharing

Data from the parent Utah suicide study has been uploaded into the NIMH Data Archive (NDA) as part of R01MH099134. This data includes demographic data from 4278 Utah suicides, electronic health records data from a subset of 3682 of these suicides, genotypes from 1317 suicides (plink file format), and exome sequence data (.bam file format) from 184 Utah suicides. The project also has a pairwise kinship table that corresponds to these suicide cases. DNA samples from 3347 Utah suicide deaths are at the NIMH Repository (RUCDR). Additional data from the study may be requested by contacting hilary.coon@utah.edu.

## Supplementary information

Supplementary Methods and Figures

Supplementary Table 1: Medical Diagnostic Phenotypes of Cases with EMR Data
