# Supplementary material for: Neurexin 1 variants as risk factors for suicide death
Source: Mol Psychiatry. 2021 Jun 25;26(12):7436–45. doi: 10.1038/s41380-021-01190-2 (PMC8709873; doi:10.1038/s41380-021-01190-2)
Supplement: Supplementary file 1 — Supplementary Methods and Figures [file 41380_2021_1190_MOESM1_ESM.docx]

**Supplementary Methods and Figure 1:** Example of nominal probability calculation for familially segregating suicide variant P469S (.docx)

**Supplementary Figure 2:** Western blot of Nrxn1α ecto-Fc proteins, immunofluorescence images of LRRTM3 and LRRTM4 binding, and graphs quantifying Fc-binding of LRRTM3 and LRRTM4 (.docx)

**Supplementary Figure 3:** Models of DAG, Nlgn, and LRRTM2 Binding and Western blot results for introducing second mutation (D1216A) at LNS6 to reveal disruptions to LNS2 binding (.docx)

**Supplementary Table 1:** Diagnostic information for cases with evidence for *NRXN1* variants. Percentages are based on 19 cases that have variants with records versus 2722 cases without the variants, with records (.xlsx)

# Supplementary methods: calculating the nominal probability of familial segregation of variants P469S and H885Y.

The nominal probability of this familial segregation was computed by considering variant frequency, the number of meioses between sharing cases, and the chance of introduction of the variant through a marry-in spouse vs. transmission at each generation. The probabilities of observing these variants on a randomly selected chromosome were estimated to be 0.0009 for P469S and 0.0008 for H885Y using the number of high/medium impact variant annotations with similar frequencies in a previous study of human genomic variation in 26,724 genes across a sample of 121,412 chromosomes1. It is estimated that there are 3,563,315 SNPs with similar functional annotations and that ~81% of these have a MAF ≤0.0033 (comparable to that of P469S), and ~72% have a MAF≤.0011, (comparable to that of H885Y). Therefore, the chance of observing a variant with the characteristics of P469S in a randomly selected gene on a randomly selected chromosome is:

(3,563,315 x 0.81) / (121,412 x 26,724) = 0.0009

and the chance of observing a variant with the characteristics of p.H885Y, similarly, is: (3,563,315 x 0.72) / (121,412 x 26,724) = 0.0008.

Segregation of P469S occurred in two different high-risk families, one with 7 meioses separating the cases, and the other with 11 meioses separating the cases. The probability of observing the segregation in these families is 2.84x10^-5^ and 8.85x10^-7^, respectively. The overall probability of familial sharing for P469S then results from the binomial probability of observing this sharing twice out of 240 high-risk families in our total sample (p=4.61x10^-5^). Segregation of H885Y was observed once in another high-risk family, with 12 meioses separating the cases and the binomial probability of this familial sharing is calculated using these same methods as 2.70x10^-4^. The overall combined probability of observing all three of these familial occurrences of rare, putatively functional *NRXN1* variants in our family sample, is, therefore, p< 1x10^-6^. We

note that this probability is not adjusted for occurrence in all 2,219 Utah suicides with genealogical data.

# Supplementary Figure 1: Probability of Familial Segregation for P469S


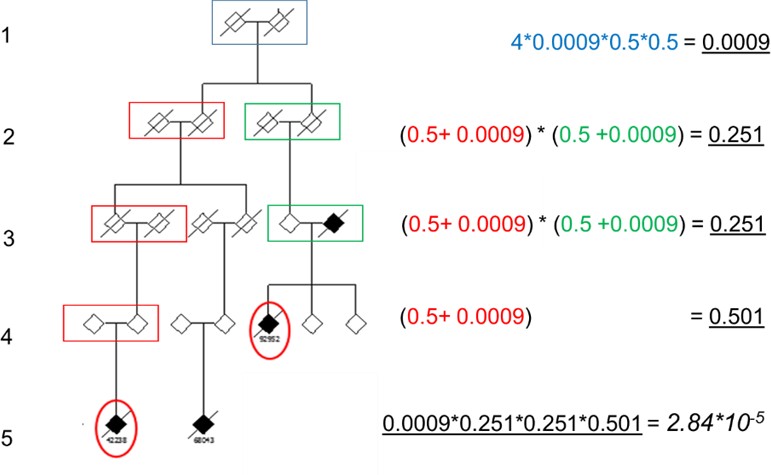


The structure of one high-risk family in which P469S segregates is shown. The separation between cases having the variant (circled red) is 7 meioses. Starting at the first generation (numbered from the top), the variant can occur in one of four ancestral chromosomes. The chance of seeing this variant on a selected chromosome computed in the supplemental section above is 0.0009. The probability of familial transmission from generation 1-2 for each offspring is 0.5. For generations 2-3, the probabilities associated with a marry-in spouse are also considered, along with the probabilities of familial transmission. For generations 2 and 3, the probability that the allele could be introduced by a marry-in spouse (0.0009) OR the probability of familial transmission (0.5) is considered for each branch of the family (color-coded red or green). For generation 4, only one branch probability exists. The total probability of familial transmission of this variant is 2.84*10^-5^.

References

1 Lek M, Karczewski KJ, Minikel E v., Samocha KE, Banks E, Fennell T, et al. Analysis of protein-coding genetic variation in 60,706 humans. Nature. 2016 Aug 17;536(7616):285–

91

# Supplementary Figure 2: Fc-Binding Assay


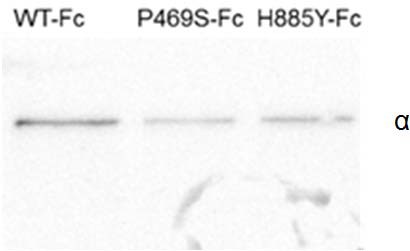

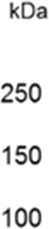
a.

-Fc

b.

c.


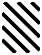

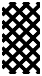

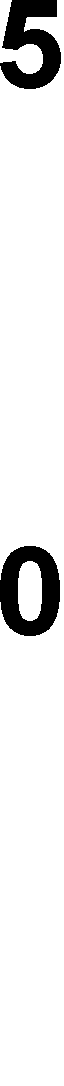

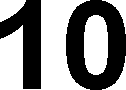

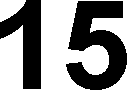

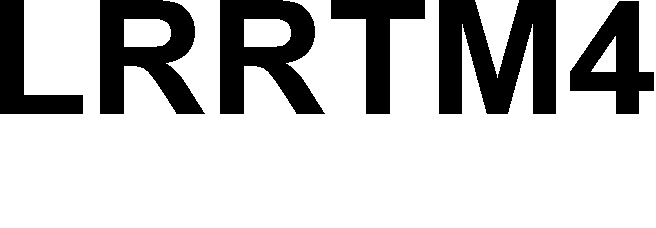

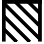

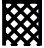

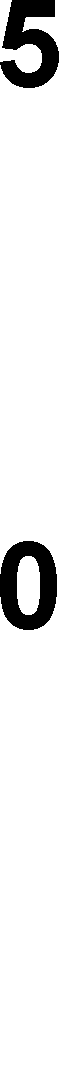

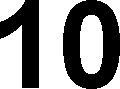

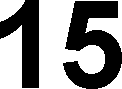

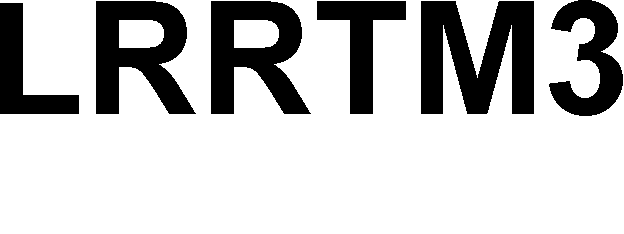


**Mean Gray Value Ratio (Fc/myc-LRRTM3)**


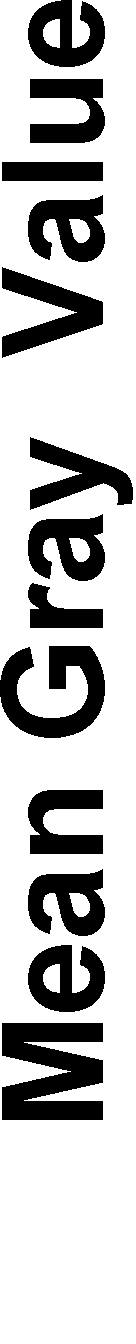

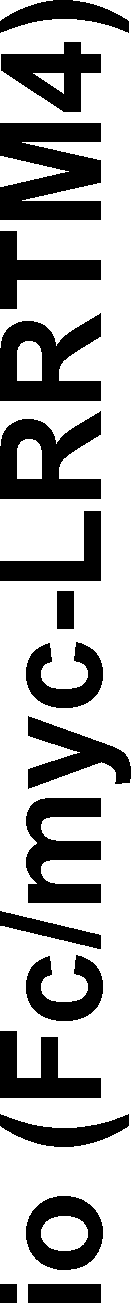

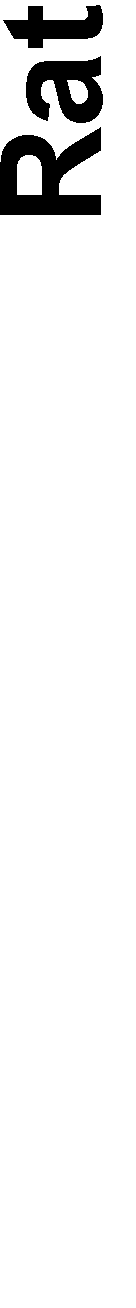

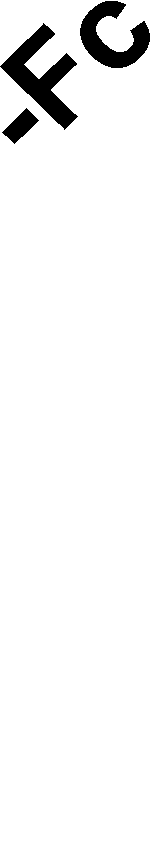

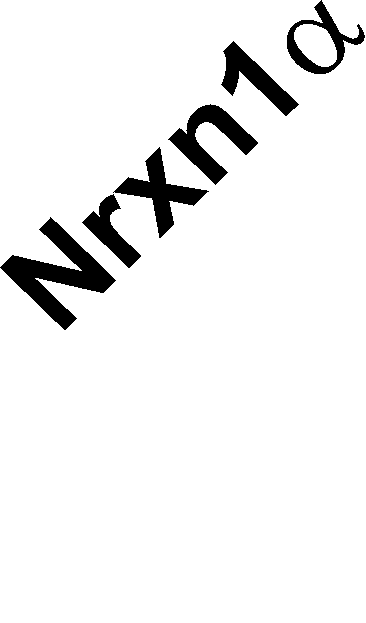

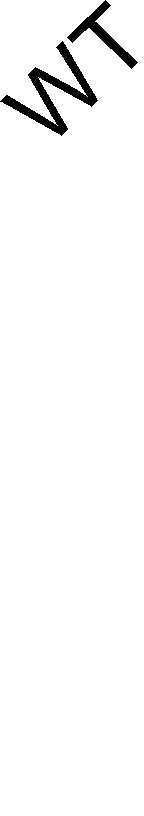

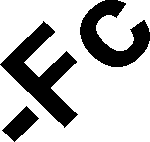

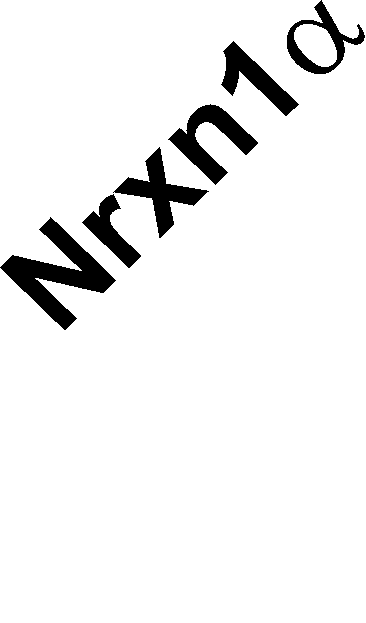

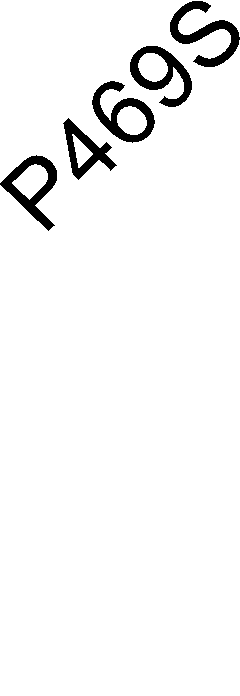

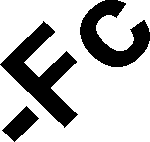

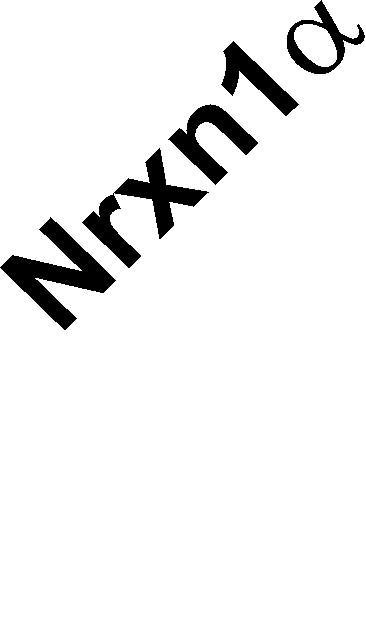

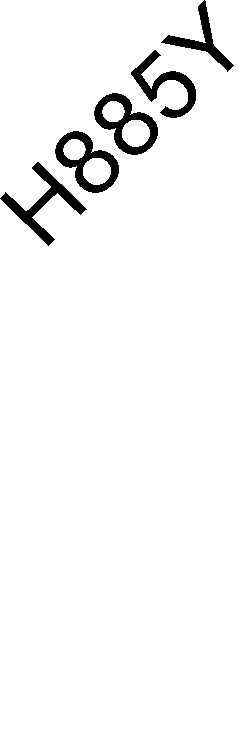

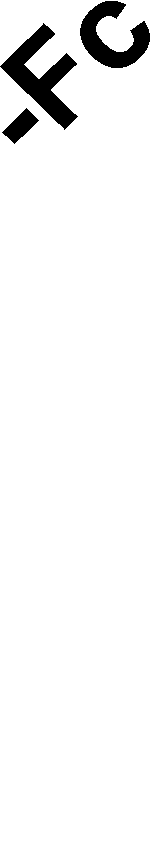

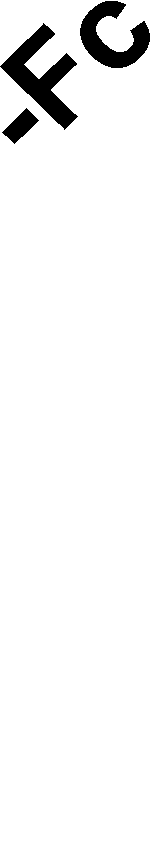

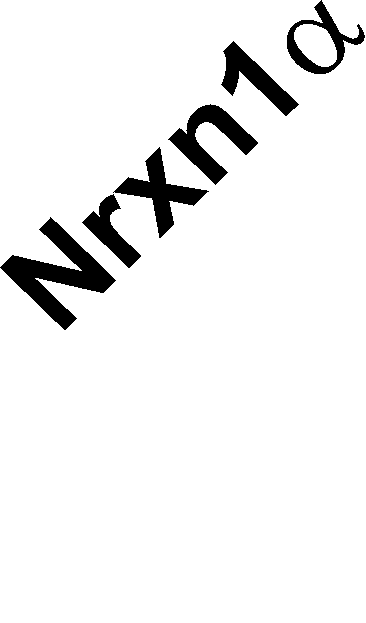

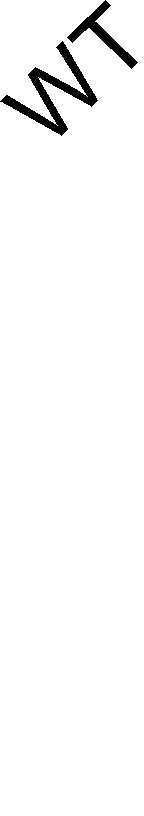

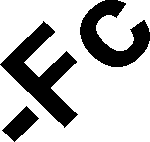

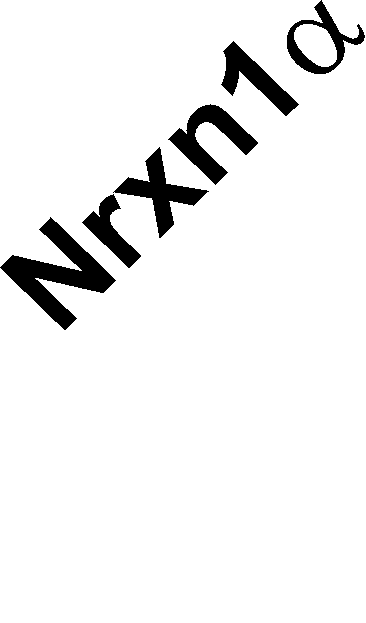

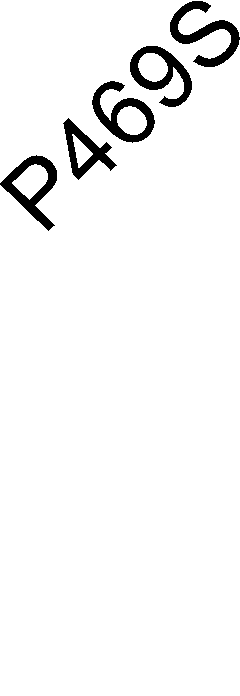

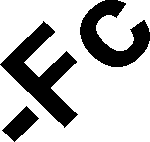

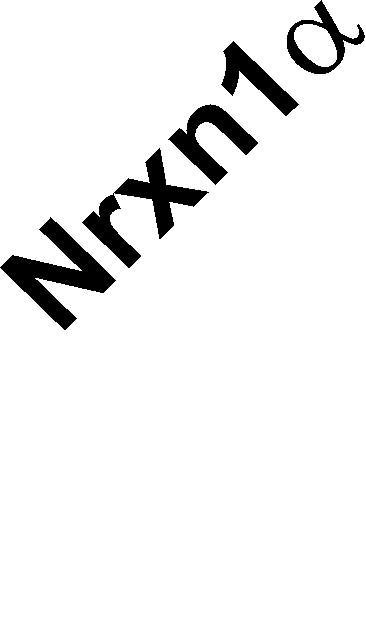

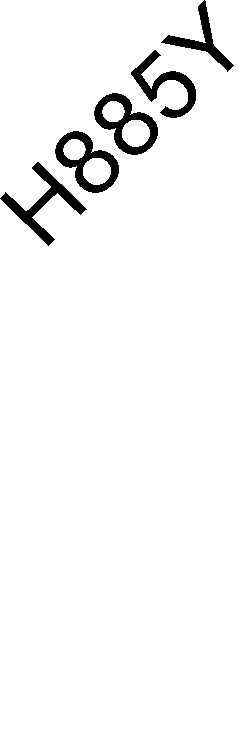

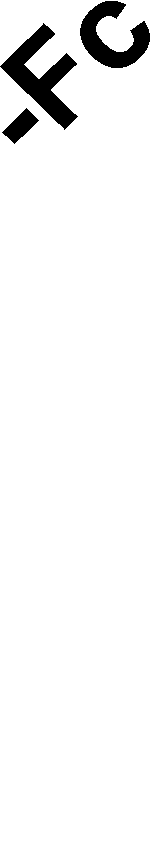


2a. Western blot showing expression of Nrxn1α-ecto-Fc proteins to be used in Fc-cell assay

2b-c. 2b. Confocal images of cell surface binding assays for LRRTM3 (left panel) and LRRTM4 (right panel). Fc-binding immunoreactivity (green), myc-tagged LRRTMs immunoreactivity (red).

2c. Quantification of 2b. For LRRTM3, (n=26 (WT); n= 25 (P469S) n=24 (H885Y) cells from 3 different cultures for each condition, ±SEM reported) For LRRTM4, (n=36 (WT); n=31 (P469S) n=42 (H885Y) cells from 3 different cultures for each condition, ±SEM reported), no significant binding differences for WT or variant conditions observed for LRRTM3 or LRRTM4.

# Supplementary Figure 3: DAG, LRRTM2, and Nlgn Binding

a.


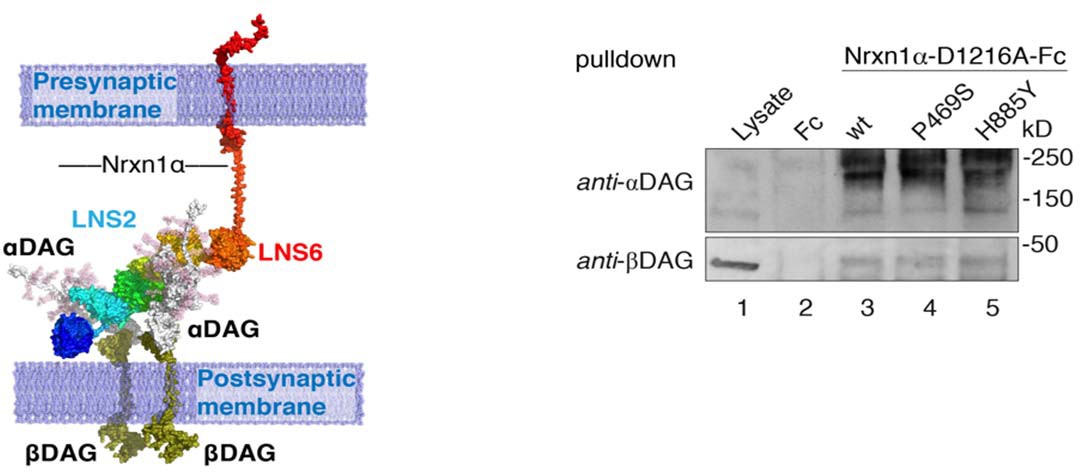


b.

c.

3a. α-DAG can bind to LNS2 and LNS6 of Nrxn1α and is predicted to have the ability to bind both sites simultaneously.

3b. To restrict the binding to LNS2, we mutated and destroyed the Ca^2+^-binding site at LNS6 (D1216A). Consequently, the amount of DAG bound to Nrxn1α-Fc is reduced. However, both mutations, P469S (lane 4) and H885Y (lane 5) allow binding similar to WT (lane 3).

3c. Structural models of Nrxn1α binding to LRRTM2 (left) and Nlgn1 (right). LRRTM2 wraps around LNS3 of Nrxn1α while binding to LNS6 of Nrxn1α. Both mutations, P469S and H885Y flank LNS3 and may release LNS2 or LNS4, respectively, from the observed rigid core structure of LNS2-LNS5, allowing a facilitated binding of LRRTM2 to LNS6. Similarly, H885 is proximal to Nlgn1 dimer binding (right). Due to the distance to Nlgn, changes at P469 are not likely to have an impact on Nlgn binding. Structures have been modeled using coordinates of Nrxn1α (PDBID:3QCW) to superimpose LNS6 with LNS of Nrxn1β in complexes of Nrxn1β/LRRTM2 (PDBID:5Z8Y) and Nrxn1β/Nlgn1 (PDBID: 3B3Q). Both complexes required repositioning of the rigid core of LNS2-LNS5 along the known hinge between LNS5 and LNS6 to prevent a steric clash.
